# Hemoptysis as the Presenting Clinical Sign of a T8-T9 Spine Fracture with Diffuse Idiopathic Skeletal Hyperostosis Changes

**DOI:** 10.1155/2016/7657652

**Published:** 2016-06-22

**Authors:** Ioannis Siasios, John Pollina, Vassilios G. Dimopoulos

**Affiliations:** ^1^Department of Neurosurgery, Jacobs School of Medicine and Biomedical Sciences, University at Buffalo, State University of New York, Buffalo, NY, USA; ^2^Department of Neurosurgery, Buffalo General Medical Center/Kaleida Health, 100 High Street, Buffalo, NY 14209, USA

## Abstract

Diffuse idiopathic skeletal hyperostosis (DISH) is a noninflammatory degenerative disease that affects multiple spine levels and, in combination with osteoporosis, makes vertebrae more prone to fractures, especially in elderly people. We describe a rare case of thoracic fracture in an ankylosed spine in which hemoptysis was the only clinical sign. The patient (age in the early 80s) presented with chest pain and a cough associated with hemoptysis. The patient had no complaints of back pain and no neurological symptoms. Computed tomography (CT) angiography of the chest revealed changes consistent with DISH, with fractures at the T8 and T9 vertebra as well as lung hemorrhage or contusion in the right lung base. CT and magnetic resonance imaging of the thoracic spine showed similar findings, with a recent T8-T9 fracture and DISH changes. The patient underwent percutaneous pedicle screw fixation from T7 to T11 and remained neurologically intact with an uneventful postoperative course.

## 1. Introduction

Diffuse idiopathic skeletal hyperostosis (DISH) is a noninflammatory degenerative disease that leads to ossification of the spinal ligaments and subsequent reduction of spinal mobility. Skeletal manifestations of DISH include ossifications and bony malformations, especially in the anterior column of the spine [1]. Other characteristic findings of the disease are extraskeletal ossifications or calcifications that could cause enthesopathy and respiratory, gastrointestinal, and neurological symptoms due to compression of surrounding tissues [1–3]. The prevalence of the disease varies from 1% to 35% and it is age dependent as the incidence increases beyond the age of 50 years [4, 5]. In addition, DISH-related changes are more often observed in men than in women in elderly populations [5].

The disease can be indistinguishable at early stages as restricted range of motion and back pain are common symptoms among elderly patients. The diagnosis is established by the involvement of four vertebral segments on imaging studies and the absence of other spine pathologies, such as disc space narrowing, and sacroiliac joint and apophyseal joint degenerative disease [3, 6]. The diagnosis of DISH is also made after spine trauma because even small forces can lead to spinal fracture [7, 8]. The main causative mechanisms are hyperextension injuries that provoke spine fractures in the less mobile, fragile regions of the diseased spine [2, 8]. The cervical segment of the spine is most affected, followed by thoracic and lumbar segments [2, 9, 10].

DISH-related spinal fractures are diagnosed by a combination of clinical symptomatology and imaging studies such as fluoroscopy, computed tomography (CT), and magnetic resonance imaging (MRI). Pathological neurological signs and localized pain in the axial spine make physicians suspicious of a spinal fracture after a hyperextension injury. We describe a unique case of a thoracic fracture associated with DISH changes in a patient who presented with the initial clinical sign of hemoptysis without neurological symptomatology.

## 2. Case Presentation

The patient (age in the early 80s) presented to the emergency department with chest pain and a cough associated with hemoptysis. The patient had experienced a fall 3 days prior to the present admission and a low velocity motor vehicle accident 2 days prior. There were no complaints of back pain and no neurological symptoms, and the patient was ambulatory without assistance.

The patient's medical history was remarkable for Barrett's esophagus, coronary artery disease, hypertension, hyperlipidemia, deep vein thrombosis, esophagitis, and pulmonary embolism. The surgical history included coronary artery bypass graft, surgical removal of a skin melanoma on the right shoulder, knee joint prosthesis implantation, and rotator cuff repair. The patient did not smoke or drink. Medication allergies included sulfa, penicillin, and erythromycin.

On examination, the patient's vital signs were within normal limits. Breath sounds were decreased bilaterally. The patient had shortness of breath and a cough. Chest pain was experienced when coughing. There was no dyspnea on exertion. There were no neurological symptoms, such as sensation disturbances, muscle weakness, or sphincter dysfunction (American Spinal Injury Association [ASIA] impairment score E).

**Figure 1:**
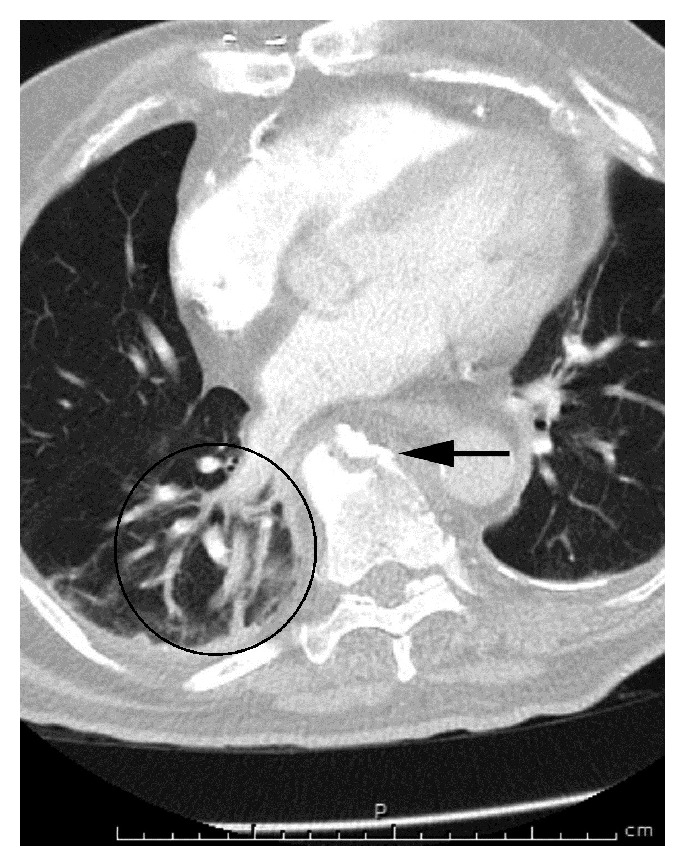
Axial CT angiogram of the chest showing a T8 vertebral fracture (*arrow*) and lung hemorrhage or possible contusion in the right lung base (*black circle*).

**Figure 2:**
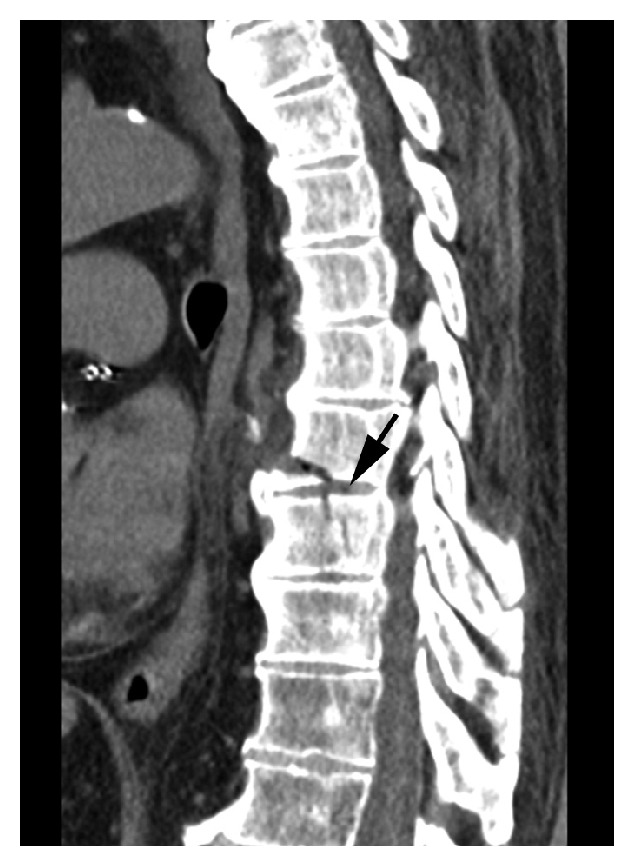
Sagittal CT scan of the thoracic spine showing an oblique fracture traversing the T8 inferior endplate, T8-T9 interspace, and posterior aspect of the T9 vertebral body (*arrow*).

**Figure 3:**
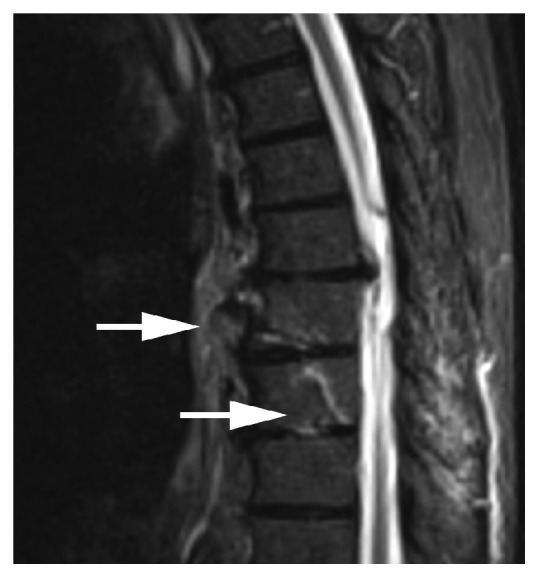
Sagittal MRI, T2 short tau inversion recovery (STIR) sequence, showing unstable acute fractures of the T8-T9 vertebrae (*arrows*).

**Figure 4:**
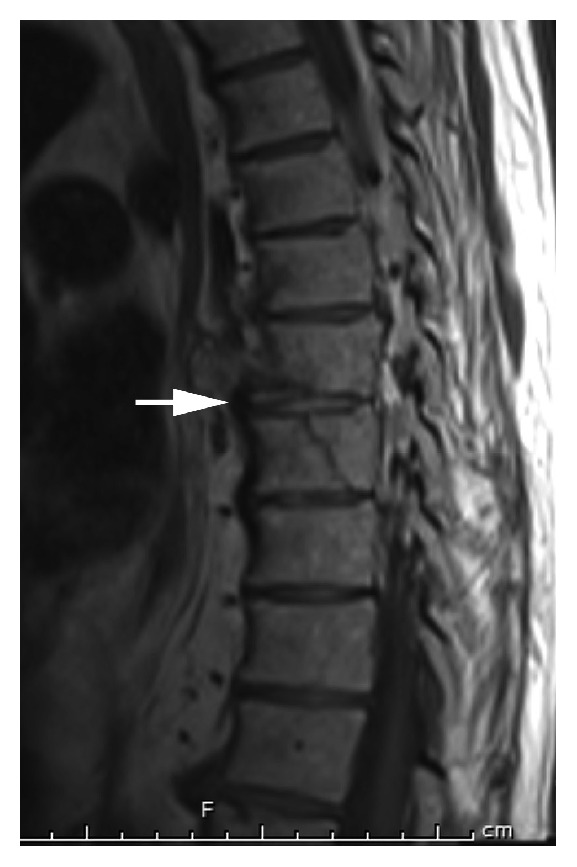
Sagittal MRI, T1 sequence, showing the T8-T9 fractures involving the anterior and middle columns with widening of the anterior disc space (*arrow*).

Blood tests were normal. A chest X-ray showed left lower lobe atelectasis but no definitive pneumonia. CT angiography of the chest showed acute fractures at the T8 and T9 vertebrae (Figure 1). In addition, the study was indicative of lung hemorrhage or a possible contusion in the right lung base. A CT scan of the cervical spine without contrast enhancement showed extensive cervical arthropathy. A CT scan of the thoracic spine showed findings compatible with a hyperextension injury with an oblique fracture traversing the T8 inferior endplate, T8-T9 interspace, and posterior aspect of the T9 vertebral body (Figure 2). MRI at this level showed similar findings, with a recent complex T8-T9 fracture with DISH changes involving the anterior and middle columns with widening of the anterior disc space (Figures 3 and 4). An associated paravertebral soft tissue swelling/hemorrhage was noted.

**Figure 5:**
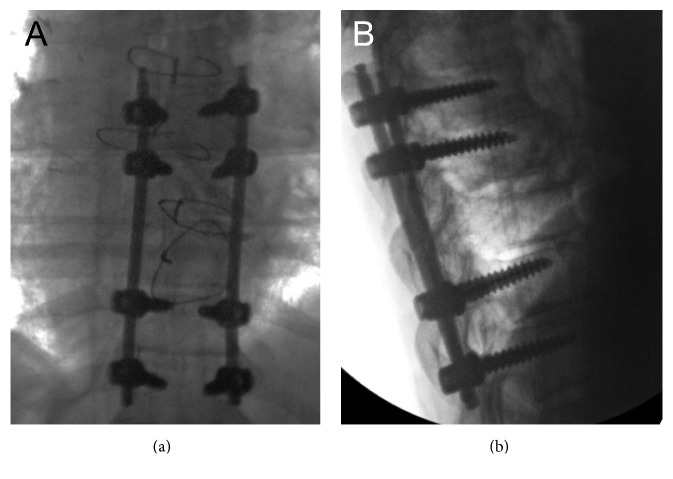
Intraoperative fluoroscopic images ((a) anteroposterior; (b) lateral) showing optimal positioning of the spinal fixation hardware as well as good alignment of the thoracic spine.

**Figure 6:**
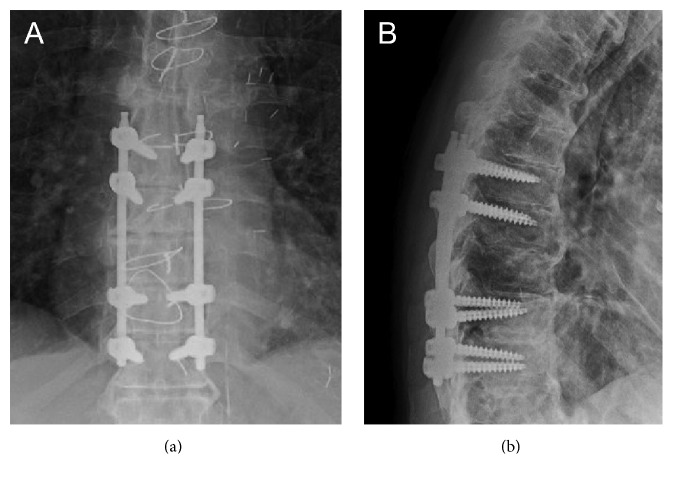
Postoperative thoracic spine X-rays ((a) anteroposterior; (b) lateral) at 3-month follow-up visit showing T7–T11 posterior fixation.

After giving informed consent, the patient underwent percutaneous pedicle screw fixation from T7 to T11 (Figure 5). The procedure was well tolerated. Postoperatively, the patient was transferred to the neurointensive care unit for further evaluation and management. A postoperative CT of the thoracic spine was performed before discharge, which revealed optimal positioning of the spinal fixation hardware as well as good alignment of the thoracic spine. The patient was discharged to a rehabilitation facility 2 days after surgery for a 1-month period. With early mobilization and physiotherapy, the lung contusion resolved 10 days after surgery. The cough completely disappeared 7 days after initiation of the rehabilitation therapy. The postoperative course was uneventful. At the time of the 3-month postoperative visit, the patient remained neurologically intact and X-rays of the thoracic spine were unremarkable (Figure 6).

## 3. Discussion

Thoracic spine injuries caused by a hyperextension mechanism are rare [11]. In the literature, there are only a few case reports and case series describing thoracolumbar fractures with DISH involvement after hyperextension injuries. Corke reported the case of a 71-year-old woman who presented with delayed neurological deterioration and back pain after experiencing minor traumatic injury [12]. This patient proved to have an upper lumbar spine fracture with ankylosing vertebral hyperostosis.

Paley et al. summarized eight cases with DISH changes encountered in their clinical practice [13]. They reported a high rate of delayed diagnosis in 3 cases (37.5%). Also, 7 (87.5%) of the patients were neurologically impaired after the fracture immediately or with the passage of time. These authors concluded that patients with DISH pathology and spinal fractures should be treated as soon as possible in an effort to restrict neurological deficit and spine instability.

Israel et al. published an interesting case of a 70-year-old woman with a T9-T10 thoracic fracture after retroperitoneal surgery in whom the spine was hyperextended and rotated [9]. The patient was paraplegic immediately after the procedure. Imaging studies revealed the thoracic fracture as well as DISH pathology in her spine.

Burkus and Denis reported four cases of DISH pathology in men who sustained thoracic fracture-dislocation subsequent to a hyperextension injury [14]. All patients were neurologically intact at the time of diagnosis. Three of them were treated with posterior fixation and one was treated conservatively with a brace. The patient who was treated conservatively had the worst outcome, with neurological deterioration and poor alignment of the spine.

Le Hir et al. described six cases of thoracic fracture through a segment of the spine ankylosed by DISH [15]. The patients were admitted to the hospital 1 to 10 days after sustaining a hyperextension injury. Only one patient had a neurological deficit, with a left-sided radiculopathy. Half of the patients became paraplegic 10 to 30 days after the traumatic event took place. Unfortunately, the surgical outcome was poor as the four patients who were treated with spinal fixation died 2 months after the procedure. The 2 other patients did not have surgery and had no residual abnormalities at the time of the 6-month follow-up examination.

Königshausen et al. described the case of a 57-year-old woman who sustained a thoracic fracture during a total hip replacement procedure [10]. Postoperatively, the patient had incomplete paraplegia. Imaging studies showed an unstable T11 vertebra fracture and DISH spinal pathology. Although the patient was treated immediately with posterior fixation, she did not improve neurologically and died 2 months after the initial procedure.

Oh et al. reported a unique case of a 62-year-old woman who sustained a lower lumbar spine fracture (L4 vertebra) associated with a hyperextension injury in a vehicle motor accident [16]. The patient experienced low back pain but had no neurological deficit. She was treated with posterior instrumentation of the lumbar spine. After the surgery, she complained of abdominal pain and voiding difficulty. Imaging studies revealed that the right ureter was impinged between the fragments of the fourth lumbar vertebra.

Caron et al. performed a retrospective study at their institution regarding the treatment of spine fractures in patients with ankylosing or DISH spine disorders. They concluded that these fractures were more common in the cervical spine [17]. Spinal cord involvement was present in 58% of the patients. A delayed diagnosis of spinal fracture was made in 21 of 112 (19%) patients, of whom 17 (81%) were neurologically compromised. Sixty-seven percent of the patients were treated with spinal fixation surgical procedures. The mortality rate was 32% and was more related to age >70 years than to other comorbidities and low-energy mechanism of injury after performing a multivariate analysis [17].

Lee reported the case of a 78-year-old woman who suffered a motor vehicle accident and was admitted to the emergency department with paraparesis [8]. Imaging studies revealed a hyperextension fracture of the first lumbar vertebra with DISH pathology as well as a spinal subarachnoid hematoma. The hematoma was drained, and the fracture was treated surgically. The patient had an unremarkable recovery.

In their review, Westerveld et al. studied spinal fractures associated with DISH and ankylosing spondylitis (AS) [18]. They concluded that 67.2% of patients with ankylosing spondylitis and 40% of those with DISH had neurologic deficits on admission, whereas delayed neurological deterioration occurred frequently. Surgical or nonoperative treatment did not alter the neurological prospects for most patients. The complication rate was 51.1% in AS patients and 32.7% in DISH patients. The overall mortality within 3 months after injury was 17.7% in AS and 20.0% in DISH cases, respectively.

Finally, in their retrospective study of spinal fractures in conjunction with hyperextension injuries of the thoracolumbar spine, Balling and Weckbach concluded that a high-energy mechanism was involved in 85.7% of cases [2]. Transdiscal injuries were more often observed in younger patients. Patients with vertebral body fractures associated with DISH were significantly older than those with transdiscal injuries. Neurological deficits were present in 22.7% of the patients after trauma. Neurological complications did not occur in low-energy injuries. The mortality rate was 20%.

All patients in the case series and reports described above experienced back pain, which was documented in the emergency room. In addition, surgical correction was reported as the first goal for the majority of these studies, although it was associated with severe complications as well as high mortality rates [2, 15, 17, 18]. That can be explained by the age and the comorbidities of the treated patients [17]. Contemporary minimally invasive spine surgical techniques allow for reductions in blood loss, pain, incision size, and duration of operative time, with a great benefit, especially for elderly patients [19]. That was the rationale for the surgical treatment in our case, which consisted of percutaneous pedicle screw fixation from T7 to T11.

Moreover, in the previously described case series, the percentage of patients having neurological impairment after a thoracic fracture with DISH characteristics ranged from 0 to 87.5% [13–15, 17, 18]. In the undiagnosed cases, the risk of neurological deterioration increased significantly after their initial presentation at the emergency department [12, 13, 15, 17]. There is only one reported case of extraskeletal symptomatology in a patient with an already known lumbar spine fracture with DISH changes [16]. In our case, the patient did not have any neurological deficit at the initial evaluation, which occurred 3 days after trauma. Also, the patient did not complain of back pain that could lead physicians to the diagnosis of fracture. The presenting clinical signs were hemoptysis and chest pain resulting from the lung hemorrhage or contusion that occurred after the hyperextension injury. Our case represents what is to our knowledge a unique extraskeletal presentation of a thoracic spine fracture associated with DISH changes.

## 4. Conclusion

Thoracolumbar fractures associated with DISH changes are usually present after hyperextension injuries, which may be of low energy. A significant percentage of affected patients are elderly and may not have neurologic deficits at the time of their presentation to the emergency department. Symptomatology may vary from pain to only extraskeletal presenting signs, like in our case, which is unique in the literature. Early diagnosis and surgical treatment could be lifesaving, as mortality and complication rates increase significantly after the initial trauma.

## Abbreviations

AS: Ankylosing spondylitis
CT: Computed tomography
DISH: Diffuse idiopathic skeletal hyperostosis
MRI: Magnetic resonance imaging.
